# All-optical control on a graphene-on-silicon waveguide modulator

**DOI:** 10.1038/s41598-017-13213-6

**Published:** 2017-10-06

**Authors:** Kelvin J. A. Ooi, Peng Chuen Leong, Lay Kee Ang, Dawn T. H. Tan

**Affiliations:** SUTD-MIT International Design Center, Singapore University of Technology and Design, 8 Somapah Road, Singapore, 487372 Singapore

## Abstract

The hallmark of silicon photonics is in its low loss at the telecommunications wavelength, economic advantages and compatibility with CMOS design and fabrication processes. These advantages are however impeded by its relatively low Kerr coefficient that constrains the power and size scaling of nonlinear all-optical silicon photonic devices. Graphene, with its unprecedented high Kerr coefficient and uniquely thin-film structure, makes a good nonlinear material to be easily integrated onto all-optical silicon photonic waveguide devices. We study the design of all-optical graphene-on-silicon (GOS) waveguide modulators, and find the optimized performance of MW cm^−2^ in optical pump intensities and sub-mm device lengths. The improvements brought by the integration of graphene onto silicon photonic waveguides could bring us a step closer to realising compact all-optical control on a single chip.

## Introduction

The thriving success of the electronics microprocessor over the past four decades is achieved with the abundant availability of silicon. However, in recent years, the increasing demand for faster processing speed and larger bandwidth have not been met, saturating at around 3 to 4 GHz^1^. This is due to limits of metallic interconnects facing signal attenuation and large power consumption at higher data rates^2^. One of the solutions is to replace them with optical interconnects. The replacement candidate, silicon photonics, is viable given its potential low cost and high compatibility with CMOS design and manufacturing process^3^. Silicon photonics also interface well with electronic transistors through power-efficient optoelectronic transceivers. Recently, Sun *et al*. has successfully demonstrated a working prototype of a chip-scale electronic-photonic system based on the silicon photonics platform^4^.

Current research is also pushing for elimination of electronic transistors for seamless integration of an all-optical computing platform^5^. In all-optical computing for silicon photonics, the nonlinear effects are used to achieve modulation. Nonlinear effects in silicon photonics have been demonstrated to process optical signals at speeds of beyond 100 Gbit/s^6^. Besides processing optical signals, nonlinear effects are also used for sensing and generation of photons for lasing and amplification. However, the inherent nonlinear Kerr effects of silicon is of the low range of 6 × 10^−18^ m^2 ^W^−1^. To achieve a reasonable level of contrast needed for optical modulation, nonlinear optical devices often need to be operated at high optical intensities and long optical device lengths. This results in high power consumption and large device footprints, which runs in contrary to the original aim to scale down device size and energy consumption in computing chips^7^.

The best way to mitigate the disadvantages of silicon nonlinear photonics is to integrate them with novel high Kerr-coefficient materials while keeping the silicon platform for its economic advantages. One of the best candidate material is graphene, which has a high Kerr-coefficient from 10^−7^ to 10^−13^ m^2 ^W^−1^ ^8–15^. Being a two-dimensional atomically thin-film material, graphene alone is not suitable to be used as photonic waveguide due to its poor optical confinement. There are efforts to design around the thin-film nature of graphene by implementing the nonlinear optical devices on graphene plasmonic waveguides^16–21^; however, due to their short propagation length of only a few micrometers^22,23^, they are not compatible with the longer-ranged photonics, only suitable to be used in an all-plasmonics platform.

Prior papers discussed graphene-based modulators placed on dielectrics only in the context of its real refractive index changes^24^, with a conclusion that graphene’s nonlinear performance is ordinary due to its high losses. However, in this paper, we show that if we take into account the nonlinear reduction in losses, or even designing extinction modulators, the performance of graphene may exceed that of silicon-on-insulator (SOI) waveguides.

Hence, by integrating graphene onto silicon waveguides, we simultaneously make use of the photonic confinement and long-range waveguiding properties of silicon-based waveguides while leveraging the high optical nonlinearities of graphene for optical switching. In this paper, we will study in detail how to integrate graphene onto a silicon waveguide and the corresponding performance improvements in all-optical switching on silicon photonic waveguides.

## Design of the graphene-on-silicon modulator

### Waveguide structure

**Figure 1:**
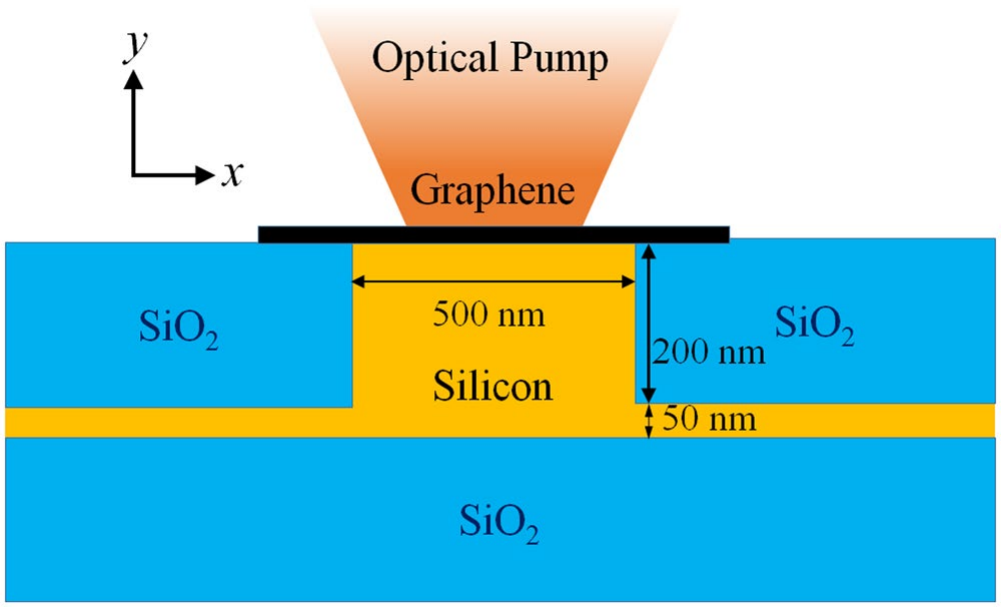
Schematic layout of the graphene-on-silicon modulator.

Figure 1 shows the schematic layout of a graphene-on-silicon (GOS) all-optical modulator. An SOI wafer is etched down into a rib cross-section of 500 nm by 200 nm, leaving a thin silicon layer of ~50 nm thickness, which has a refractive index of 3.48. The sides of the rib is then filled with SiO_2_ with refractive index of 1.44. A graphene sheet with atomic thickness of ~0.3 nm is then laid on top of the waveguide, with a sheet width large enough to cover the fundamental mode-area of the silicon rib. Our choice of adopting the SOI rib waveguide is based on extensive studies on its good confinement and low waveguide propagation and bending losses^25,26^. On top of that, this structure exposes the top waveguide surface, which allows easy placement of graphene close to the mode-propagation area, allowing graphene to have maximum interaction with the fundamental optical mode. To control nonlinear optical response of the modulator, an optical pump is shined directly on the graphene surface to modulate the Kerr refractive index.

To quantify the interaction of the waveguide mode with the graphene sheet, we define the effective mode area of graphene, *A*_*geff*_, which is written as

1 $${A}_{geff}=\frac{{(\iint {|E|}^{2}dxdy)}^{2}}{{\iint }_{graphene}\frac{1}{2}{|{E}_{x,z}|}^{4}dxdy}$$

where the electric-fields in the numerator are integrated over the whole optical waveguide structure, while in the denominator, only the in-plane electric-fields across the graphene sheet are evaluated. This takes into account the fact that only the in-plane electric-fields can perturb the Kerr nonlinearities due to the two-dimensional nature of graphene.

**Figure 2:**
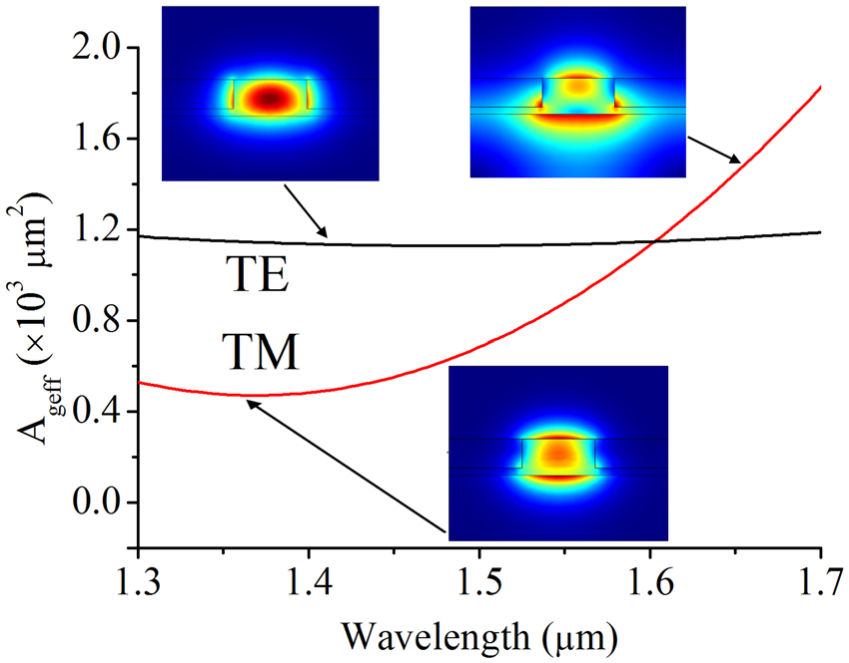
Effective mode area of graphene. The electric-field maps for selected TE and TM modes are shown in the inset.

Solutions of *A*_*geff*_ are easily found with the help of mode simulations in COMSOL. Here, we shall limit our study to only the fundamental TE and TM modes of the silicon rib waveguide. Figure 2 shows how the *A*_*geff*_ of the TE and TM modes vary with wavelength in the range from 1.3 to 1.7 µm. The *A*_*geff*_ of the TE mode is relatively constant at around 1.2 × 10^3^ µm^2^. On the other hand, the *A*_*geff*_ of the TM mode rises significantly with the increase in wavelength. A closer inspection on the electric-fields on the TM mode reveals their asymmetrical distribution at the top and bottom interfaces of the waveguide. At longer wavelengths, the electric-fields are increasingly distributed more to the bottom of the waveguide, thus the mode interacts less with the graphene sheet.

### Linear and nonlinear optical properties of grapheme

**Figure 3:**
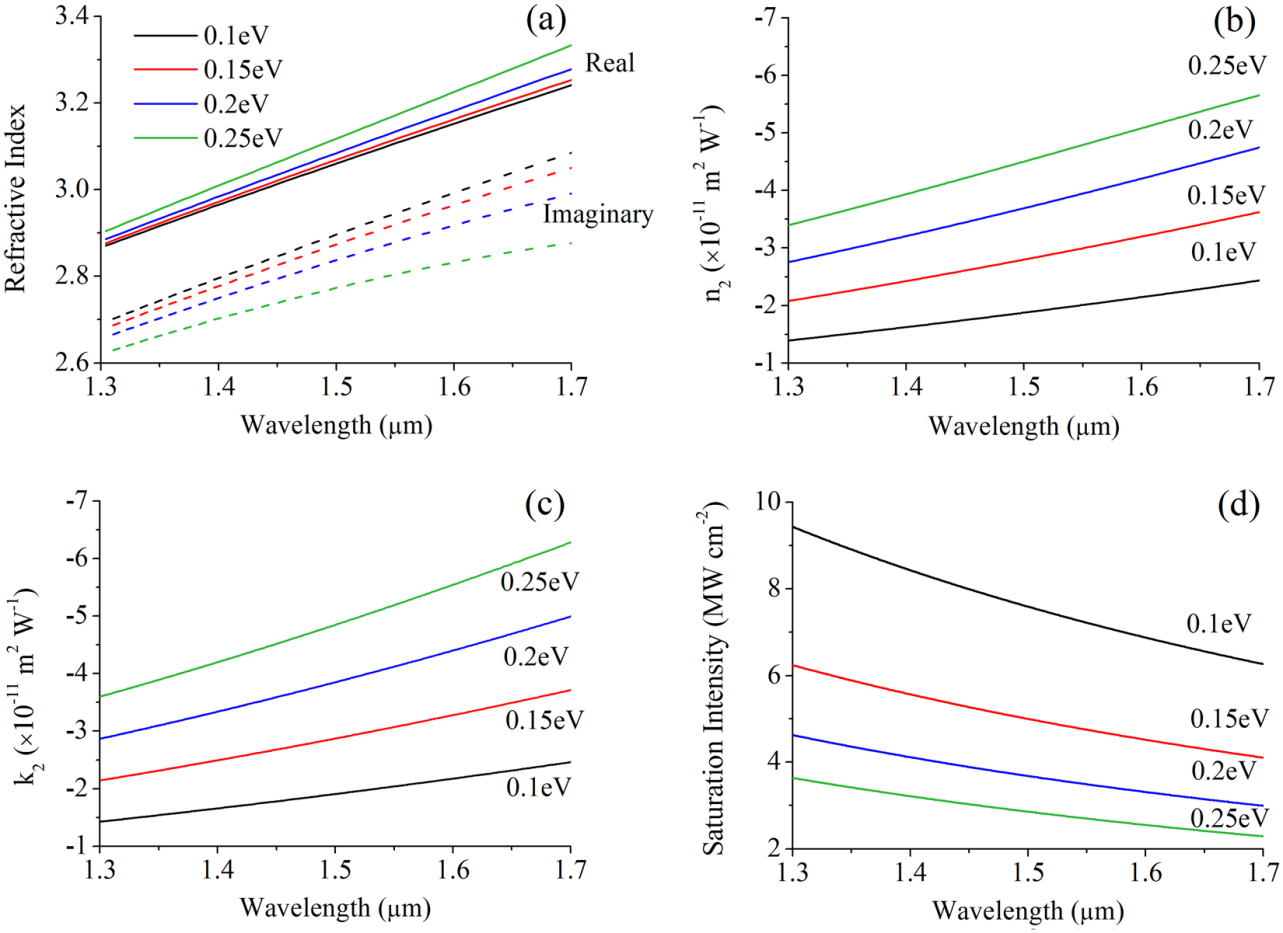
Linear and nonlinear optical properties of graphene. (**a**) Linear refractive indices. (**b**) Nonlinear refractive indices. (**c**) Nonlinear extinction coefficients. (**d**) Saturation intensity.

The linear graphene optical conductivity is described by the Kubo formula^27^

2 $$\begin{array}{rcl}{\sigma }^{(1)}(\omega ) & = & \frac{i{e}^{2}(2{k}_{B}T)}{\pi {\hslash }^{2}(\omega +i{\nu }_{1})}\{\frac{{E}_{F}}{2{k}_{B}T}+\,\mathrm{ln}[2\,\exp (-\frac{|{E}_{F}|}{{k}_{B}T})+1]\}\\  &  & +\frac{i{e}^{2}}{4\hslash }\{0.5+{\tan }^{-1}[\frac{\hslash (\omega +i{\nu }_{2})-2|{E}_{F}|}{2{k}_{B}T}]\\  &  & -\frac{i}{2\pi }\,\mathrm{ln}(\frac{{[\hslash (\omega +i{\nu }_{2})+2|{E}_{F}|]}^{2}}{{[\hslash (\omega +i{\nu }_{2})-2|{E}_{F}|]}^{2}+{(2{k}_{B}T)}^{2}})\}\end{array}$$

which is a function of the radian frequency *ω*, relaxation frequencies *ν*_1_ and *ν*_2_^23,28^, and Fermi-level *E*_*F*_ at room temperature *T* = 300 K. The optical conductivity can be used to derive the refractive index of graphene using

3 $${n}_{graphene}(\omega )=\sqrt{1+\frac{i{\sigma }^{(1)}(\omega )}{{\varepsilon }_{0}\omega {d}_{eff}}}$$

where *d*_*eff*_ is the graphene’s layer thickness approximated to 0.3 nm. The refractive index of graphene is plotted with wavelength in Fig. 3(a). Under low doping conditions (*E*_*F*_ < 0.4 eV), graphene is a lossy dielectric in the wavelength range from 1.3–1.7 µm. Graphene-on-substrate has a natural substrate doping of around 0.1–0.2 eV, thus we will base our device analysis around this range of Fermi levels^29^.

Meanwhile, the nonlinear optical conductivity of graphene has been obtained from theory and experiments, which ranges from 10^−11^–10^−13^ m^2^W^−1^ around 1.55 µm^8–15^. Graphene’s nonlinear conductivity is calculated using semiconductor Bloch equations based on graphene’s tight-binding model by Cheng *et al*.^14,15^. These values can be transformed to the nonlinear refractive index using^30^

4a $${n}_{2}=\frac{3}{4{\varepsilon }_{0}c({{n}_{graphene}}^{2}+{{k}_{graphene}}^{2})}\,[{\chi }_{R}^{(3)}+\frac{{k}_{graphene}}{{n}_{graphene}}{\chi }_{I}^{(3)}]$$

4b $${k}_{2}=\frac{3}{4{\varepsilon }_{0}c({{n}_{graphene}}^{2}+{{k}_{graphene}}^{2})}\,[{\chi }_{I}^{(3)}-\frac{{k}_{graphene}}{{n}_{graphene}}{\chi }_{R}^{(3)}]$$

where *n*_*graphene*_ and *k*_*graphene*_ are the real and imaginary linear refractive indices of graphene, and ${\chi }^{(3)}=i{\sigma }^{(3)}/{\varepsilon }_{0}\omega {d}_{eff}$ is the nonlinear Kerr susceptibility, partitioned to the real (${\chi }_{R}^{(3)}$) and imaginary (${\chi }_{I}^{(3)}$) parts respectively. We used theoretically calculated values of graphene’s Kerr coefficientfrom 1.3–1.7 µm for different *E*_*F*_ as shown in Fig. 3(b) and (c). In this spectrum, it is found that the Kerr coefficient does not vary much for *E*_*F*_ from 0.1–0.25 eV, much unlike the behavior in the midinfrared where the low *E*_*F*_ regime has nonlinearities higher by a few orders^21^.This can be qualitative explained by the fact that at low-frequency regime the optical response is dominantly contributed by intraband process which has a stronger Fermi level and frequency dependence while at high-frequency regime the dominating interband process has a much weaker frequency and Fermi level dependences^12,13^. At 1.55 µm, the Kerr coefficient of graphene is between 2–5 × 10^−11^ m^2^W^−1^, which is seven orders higher than silicon’s Kerr coefficient of 6 × 10^−18^ m^2 ^W^−1^.

It is noted that the magnitude of graphene’s real and imaginary Kerr coefficients is negative. The impact of the negative imaginary Kerr coefficient, also called the saturable loss^28^, is to lower the overall propagation loss of graphene after modulation. This would augment the phase-modulation performance of the graphene-on-silicon waveguide, and at the same time enable the design of an extinction-based modulator.

Finally, we also take into account the saturation intensity of the nonlinear Kerr modulation using the standard experimental definition

5 $${k}_{graphene}+{k}_{2}{I}_{sat}=\frac{{k}_{graphene}}{2}$$

The saturation intensities are in the range of a few MW cm^−2^ as shown in Fig. 3(d).

## Modulation performance and Discussion

### Nonlinear waveguide indices

To obtain the effective nonlinear modulation of the GOS waveguide would require analysis of the entire optical mode propagation in the waveguide structure. This is easily achieved through mode simulations in COMSOL, where the effective refractive indices before and after the optical modulation are analysed. In order to give a more accurate picture of the nonlinear performance contributed by graphene, we have omitted the nonlinear refractive index of silicon from our simulations. The difference between the values, *Δn* and *Δk*, would give us the effective nonlinear refractive index through

6a $${n}_{total}={n}_{eff}+\Delta n={n}_{eff}+{n}_{2}I$$

6b $${k}_{total}={k}_{eff}+\Delta k={k}_{eff}+{k}_{2}I$$

Here, it is noted that *n* and *k* denote the effective waveguide indices instead of the material indices. Also, the optical intensity, *I*, has the implicit term of $I/(1+I/{I}_{sat})$ to take into account the limits of saturation intensity on the nonlinear modulation. Therefore, to get the correct waveguide*n*_2_ and *k*_2_ values, we use *I* = 1 kW cm^−2^ in the simulation, which is significantly less than *I*_*sat*_.

**Figure 4:**
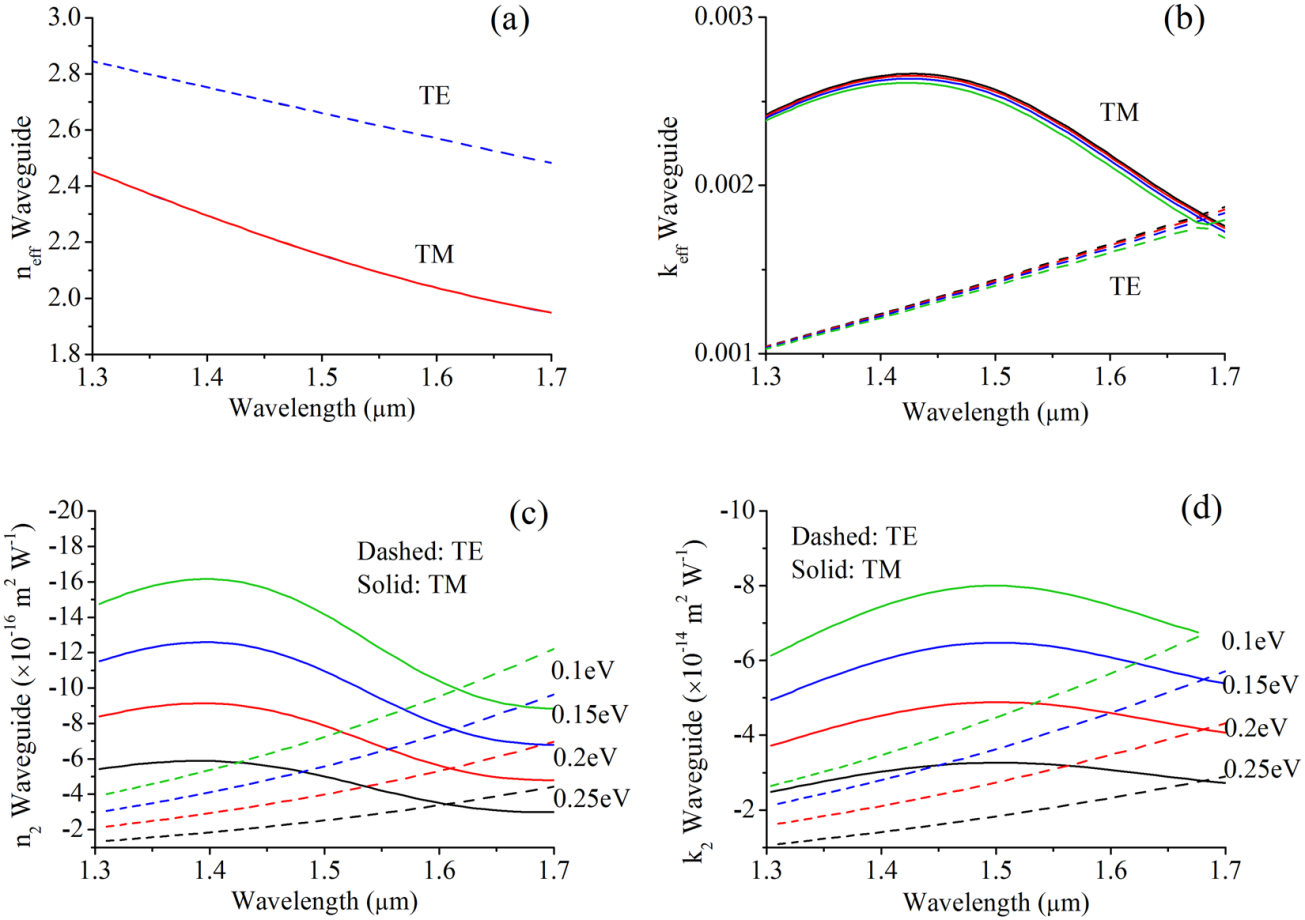
(**a**) Real linear refractive indices and (**b**) linear extinction coefficients of the GOS waveguide. (**c**) and (**d**) are the real and imaginary nonlinear effective indices of the GOS waveguide.

The linear effective waveguide indices of the GOS waveguide are shown in Fig. 4(a) and (b). Since the optical mode’s interaction with the graphene layer is limited, the variation in graphene’s refractive indices across Fermi-levels have little influence on the effective waveguide indices. Meanwhile, in Fig. 4(c) and (d) we show the extracted values of the waveguide *n*_2_ and *k*_2_ for both the fundamental TE and TM modes. The slight increase of the waveguide nonlinear indices with wavelength for the TE mode is in line with trend for the nonlinear material indices for graphene. For the TM mode, however, the waveguide nonlinear indices drop off at the longer wavelengths due to diminished optical interaction with graphene, which arose from the asymmetric distribution of the electric-fields as has been shown before in Fig. 2. Overall, the TM mode has higher waveguide nonlinear indices compared to the TE mode, due to the higher electric-field intensities at the SOI waveguide surface-boundary in contact with graphene.

Another important point of observation is that the magnitude of the waveguide *k*_2_ is at least an order higher than that of *n*_2_, even though the material *n*_2_ and *k*_2_ magnitudes are almost the same. This is intuitively understood by looking at the linear material properties of both silicon and graphene. The refractive index of silicon is large (*n*_*Si*_ = 3.48) and since the optical mode occupies the silicon to a large spatial extent, graphene’s nonlinear change in material refractive index have little influence on the overall effective waveguide refractive index. This is not the case for the extinction coefficient, as while it is negligible for silicon in the 1.3–1.7 µm spectrum, it is quite substantial for graphene (*k*_*graphene*_ ~3). Hence, any nonlinear change in graphene’s extinction coefficient would show up prominently in the effective waveguide losses. The implication of this result is that the GOS nonlinear waveguide is better suited to be designed as an extinction-based modulator, as we will understand better through further analysis below.

### Nonlinear parameters

Another way to quantify the nonlinearity of a waveguide that also takes into account the effective optical mode area is through its nonlinear parameters. The common definition of the nonlinear parameter containing the real nonlinear index is given as

7a $${\gamma }_{n}=\frac{2\pi {n}_{2}}{{\lambda }_{0}{A}_{geff}}$$

where *λ*_0_ is the free-space wavelength. We can also define the nonlinear parameter containing the nonlinear extinction coefficient through modifying the expression to

7b $${\gamma }_{k}=\frac{4\pi {k}_{2}}{{\lambda }_{0}{A}_{geff}}$$

**Figure 5:**
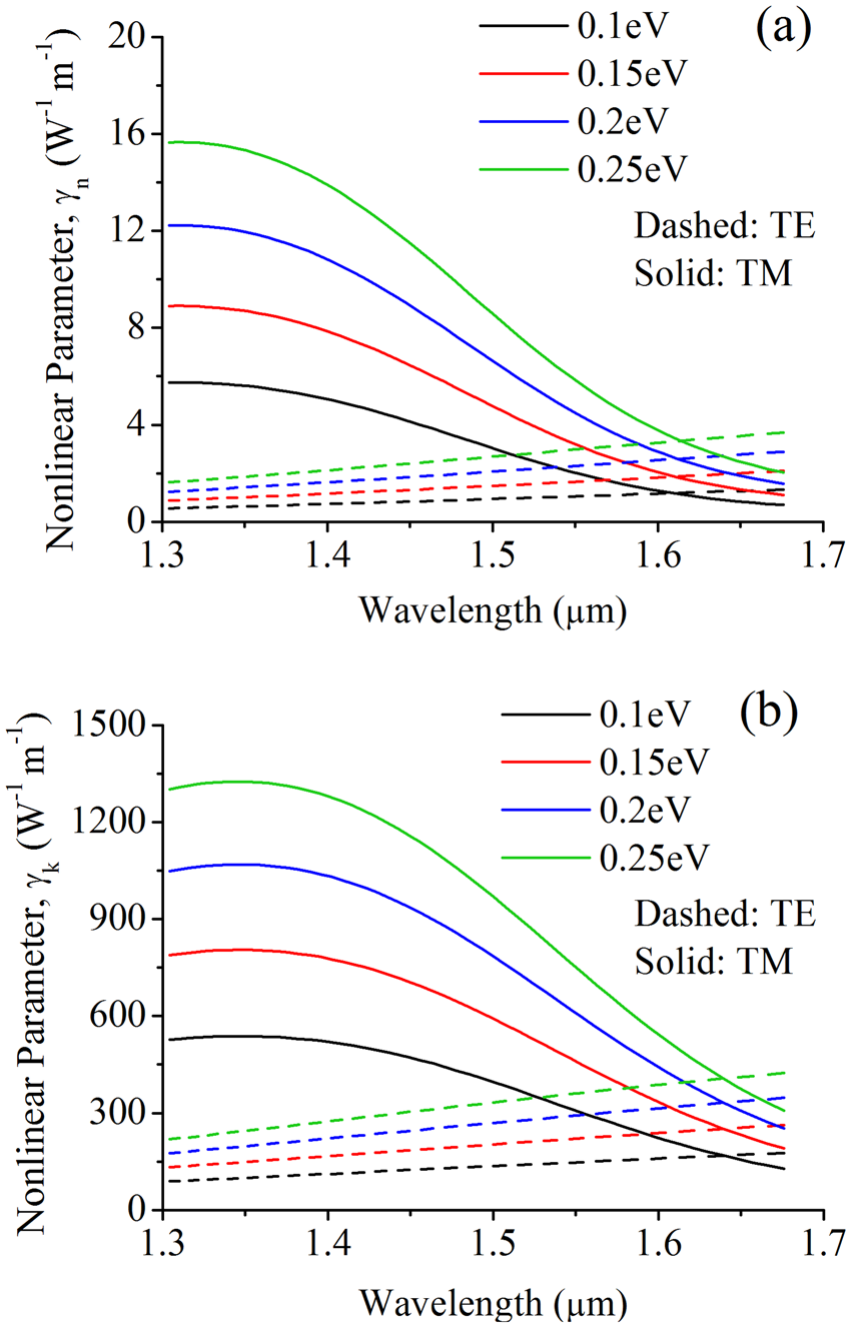
(**a**) Real γ_n_ and (**b**) imaginary γ_k_ nonlinear parameters of the GOS waveguide.

The nonlinear parameters, both in units W^−1^ m^−1^, are plotted in Fig. 5. Although previously the effective nonlinear waveguide index of the GOS waveguide is shown to be at least two orders higher than that of a standard SOI waveguide, here *γ*_*n*_ is expected to be low since the optical mode interaction with graphene is minute. In Fig. 5(a), it is shown to be in the range of 1–3 W^−1^ m^−1^ for the TE mode and an average of 3–10 W^−1^ m^−1^ for the TM mode, which is far lower than the typical values for SOI waveguides in the order of 100 W^−1^m^−1^. However, an interesting case is observed for *γ*_*k*_, which has values of more than two-orders higher than its real counterpart, in the range of 150–400 W^−1^ m^−1^ (or 650–1700 dB W^−1^ m^−1^) for the TE mode and an average of 300–1000 W^−1^ m^−1^ (or 1300–4300 dB W^−1^ m^−1^) for the TM mode, as shown in Fig. 5(b). This presents a huge potential for GOS waveguides to be used as an all-optical nonlinear extinction modulator with unlimited switching contrast.

### Nonlinear switching design and performance

Here we shall study the construct of two types of nonlinear GOS modulator, which are the phase and extinction modulators respectively. For phase modulator, the objective is to perform a π-phase shift for constructive/destructive interference in a Mach-Zehnder interferometer (MZI), so as to achieve maximum contrast between the on/off states. It is also assumed that only one arm of the MZI is covered with graphene to minimize the device’s insertion loss. The maximum phase-shift of such a modulator is given as

8a $$\Delta {\phi }_{\max }=\frac{2\pi }{{\lambda }_{0}}{n}_{2}I\cdot {L}_{eff-nonlinear}(I)$$

Here, *I* implicitly carries the usual saturation terms, while the nonlinear effective length of the waveguide will lengthen according to the optical pump intensity due to the saturable absorption effect. The use of the nonlinear effective waveguide length ensures that the output signal intensity from the GOS waveguide is high enough to combine interferometrically with the signal from the reference arm.

On the other hand, for the extinction modulator we can similarly write the maximum extinction change as

8b $$\Delta {\alpha }_{\max }=\frac{4\pi }{{\lambda }_{0}}{k}_{2}I\cdot {L}_{eff-nonlinear}(I)$$

Here, since the extinction is reduced with optical intensity, the nonlinear effective waveguide length is also defined for the on state, i.e. after the nonlinear switching occurs.

**Figure 6:**
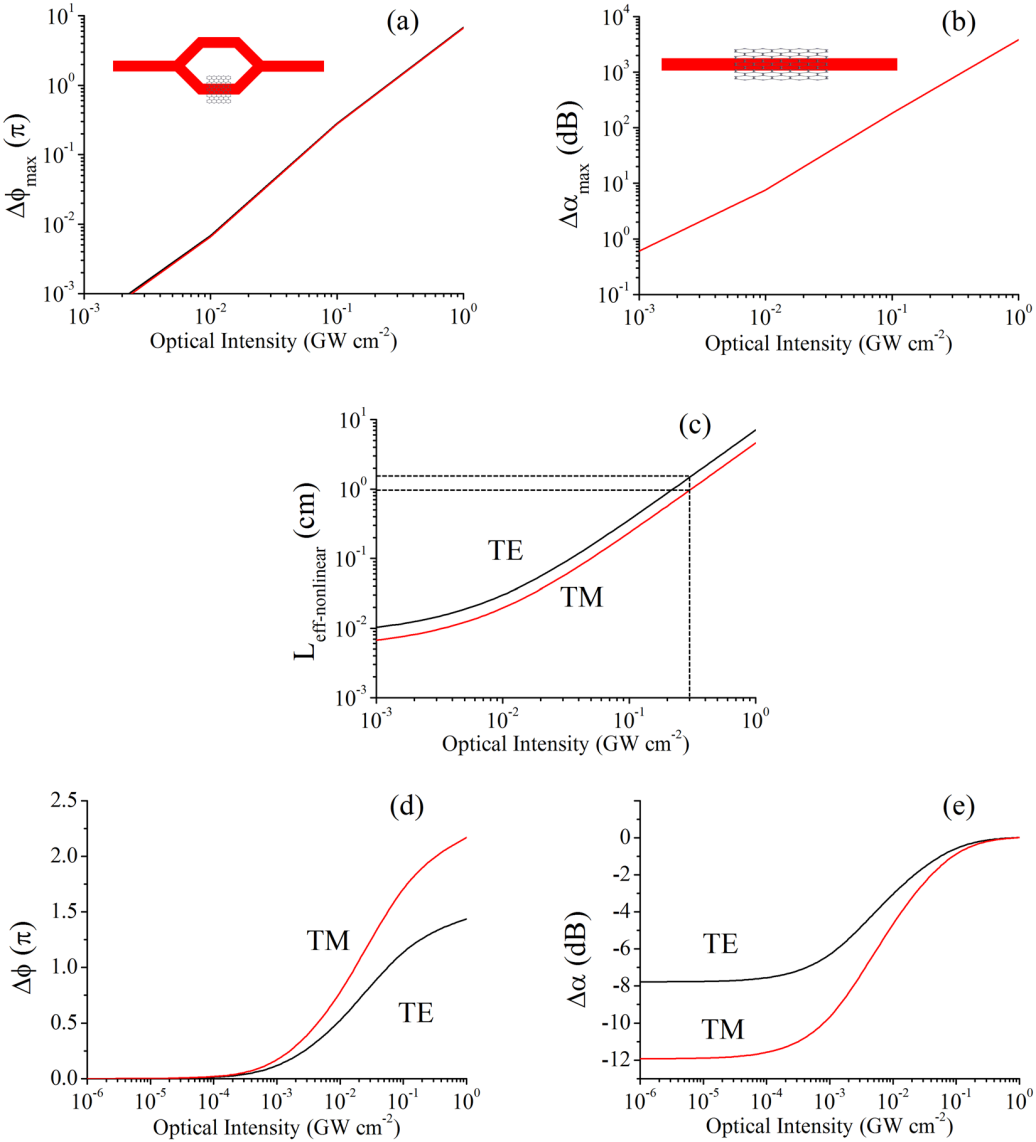
Maximum (**a**) phase-shift and (**b**) extinction change of the GOS modulator with respect to optical pump intensity. (**c**) Nonlinear effective waveguide length of the GOS modulator. (**d**) Phase-shift of a 1.5 cm GOS waveguide phase modulator. (**e**) Extinction change of a 0.14 mm GOS waveguide extinction modulator.

To illustrate the design steps of the nonlinear modulators, we first choose an example GOS waveguide, with parameters of graphene *E*_*F*_ = 0.1 eV and operating wavelength of 1.55 µm. For an MZI phase modulator, the optical signal is split equally to both arms, and only one of the arms is coated with the graphene layer. To determine the minimum optical pump intensity required for the device to perform a π-phase shift, we plot out *∆ϕ*_*max*_ in Fig. 6(a). From the plot, we find that π-phase shift is only realized at minimum *I ~* 0.3 GWcm^−2^, for both the TE and TM modes. The corresponding nonlinear effective waveguide length for these two modes, read from the *L*_*eff-nonlinear*_ plot in Fig. 6(c), are approximately 1.5 cm and 1 cm respectively. Thus, to accommodate the switching of both modes in the waveguide, we pick the GOS waveguide length as 1.5 cm. The corresponding variation of the phase-shift with intensity is plotted in Fig. 6(d). In the off-state, the GOS waveguide arm will undergo huge waveguide losses, and thus the output intensity comes only from the reference arm, representing a 3 dB loss. After nonlinear modulation, the output from the GOS arm undergoes a π-phase shift and the loss is reduced simultaneously. The output from the GOS arm can now interfere destructively with the output from the reference arm to switch off the optical signal.

On the other hand, the design of the extinction modulator is more straightforward. In a similar fashion, we plot *∆α*_*max*_ in Fig. 6(b) to find the minimum optical pump intensity for switching contrast of more than 3 dB. It is found that this could occur for optical intensities as low as 10 MWcm^−2^, and the corresponding *L*_*eff-nonlinear*_ for the TE and TM modes are 0.22 mm and 0.14 mm respectively, which are at least 70 times shorter than the phase modulator. To accommodate switching of both modes, we pick the shorter waveguide of the two (0.14 mm) to minimize insertion loss for the TM mode. Finally, Fig. 6(e) shows the variation of the signal intensity (in unit dB) with the optical pump intensity. With reference to the 10 MW cm^−2^ pump intensity, the TE mode switches from −8 dB to −3 dB, representing a contrast of 5 dB, while the TM mode switches from −12 dB to −4 dB, a contrast of 8 dB. There is also the possibility of constructing even shorter waveguides by allowing an increase to the pump intensity.

A quick performance evaluation could be made for the nonlinear GOS waveguide devices against a typical nonlinear SOI waveguide. The typical 500 nm × 200 nm SOI waveguide have a nonlinear refractive index of 6 × 10^−18^ m^2 ^W^−1^, and *A*_*eff*_ of 2 × 10^−13^ m^2^ for the fundamental TE mode, which transcribes to a nonlinear parameter of ~100 W^−1^ m^−1^ at the telecommunications wavelength^6,7^. Using a waveguide length of 1.5 cm, the required optical intensity to modulate a π-phase shift is 0.86 GW cm^−2^. The nonlinear GOS phase modulator, in comparison, require a slightly lower optical intensity at 0.3 GW cm^−2^.

In stark contrast to the nonlinear GOS phase modulator, the nonlinear GOS extinction modulator compares very favorably to the typical nonlinear SOI modulator. The nonlinear GOS extinction modulator has a device length 70 times shorter at 0.14 mm and the required optical pump intensity is 80 times lower at 10 MW cm^−2^.

Throughout the discussion of our simulated results, it is assumed that the optical intensity is low (<10 GW cm^−2^), and the saturable absorption phenomenon dominates. However, for higher optical intensities above 10 GW cm^−2^, it is possible that the two-photon absorption (TPA) process will take over and cause an increase in device optical loss with increasing optical intensity, as evidenced in ref.^31^. Also, the temporal optical response of graphene is ultrafast, possibly reaching Petahertz (PHz) or sub-femtosecond (sub-fs) timescales from a very recent study^32^.

Overall, our results show that the GOS extinction modulator offer the best modulation performance. It operates on optical intensities 80 times lower, and device lengths 70 times shorter, when compared to the SOI modulator. The packaging of illumination outlets to the graphene surface may be a challenge, and may be achieved through structures such as through-silicon-vias (TSV) in direct contact with graphene. Fabrication of such structures may be possible in the near future with advancement of technologies such as wafer transfer and embedding of graphene^33,34^.

## Conclusion

We have studied the use of graphene to enhance the nonlinear properties of an SOI photonic waveguide. Graphene has a giant nonlinear Kerr coefficient which enables all-optical modulation at low optical intensities. Also, its existence as a thin film makes it easily integrated onto current existing SOI waveguide platforms, which is a highly desirable structural property, in contrast to other bulk nonlinear materials where integration requires etching and deposition, or hybrid integration via evanescent coupling to a separate waveguide platform.

The GOS waveguide’s only drawbacks are the high linear absorption and limited access to the photonic waveguide modes. Nonetheless, the high linear absorption of graphene has already been compensated by its high saturable absorption at very low optical intensities in the order of 10 MW cm^−2^. Direct irradiation of the optical pump on the graphene surface requires very low optical intensities to perform modulation with very high switching contrast of at least 5–8 dB. In addition, GOS waveguide lengths are by far shorter than standard SOI nonlinear waveguides. These performance improvements brought by the integration of graphene on nonlinear silicon photonic waveguides could pave the way for more compact and low-powered all-optical devices for chip-scale integration.

## Methods

To obtain the effective nonlinear waveguide Kerr index, two sets of effective waveguide indices are obtained using two-dimensional MODE simulations in COMSOL application. In the first set, graphene’s linear refractive index is used in the simulation. In the second set, graphene’s nonlinear change in refractive index for an optical pump intensity, *I* = 1 kW cm^−2^, is separately calculated using data from refs^14,15^. The calculated value is added to the linear refractive index for simulation. The difference between the effective indices from the two sets of simulation, *n*_*eff1*_ and *n*_*eff2*_, would give us the effective index change

9 $${\rm{\Delta }}{n}_{eff}={n}_{eff2}-{n}_{eff1}$$

The effective nonlinear waveguide Kerr index is then easily found using

10 $${n}_{2-eff}={\rm{\Delta }}{n}_{eff}/I$$

### Data availability

All data generated or analyzed during this study are included in this published article.
